# What Teens Hear and How They React: Adolescent Perspectives on Substance Use, Sexual Risk, and Sexual Violence Prevention in Primary Care

**DOI:** 10.3390/bs16040492

**Published:** 2026-03-26

**Authors:** Daniel W. Oesterle, Leigh E. Ridings, Elizabeth M. Wallis, Sharon Levy, Kenneth J. Ruggiero, Debra Kaysen, Holly C. Gooding, Carla Kmett Danielson, Amanda K. Gilmore

**Affiliations:** 1 Department of Psychological Sciences, Purdue University, West Lafayette, IN 47907, USA; 2 Department of Psychiatry & Human Behavior, Warren Alpert Medical School of Brown University, Providence, RI 02903, USA; 3 College of Nursing, Medical University of South Carolina, Charleston, SC 29425, USAruggierk@musc.edu (K.J.R.); 4 Department of Psychiatry & Behavioral Sciences, Medical University of South Carolina, Charleston, SC 29425, USA; wallis@musc.edu (E.M.W.); danielso@musc.edu (C.K.D.); 5 Department of Pediatrics, Medical University of South Carolina, Charleston, SC 29425, USA; 6 Division of Addiction Medicine, Boston Children’s Hospital, Boston, MA 02130, USA; sharon.levy@childrens.harvard.edu; 7 Department of Pediatrics, Harvard Medical School, Harvard University, Boston, MA 02115, USA; 8 Department of Psychiatry & Behavioral Sciences, Stanford University Medical Center, Stanford, CA 94305, USA; dkaysen@stanford.edu; 9 Department of Pediatrics, Emory University School of Medicine, Atlanta, GA 30322, USA; holly.gooding@emory.edu; 10 Department of Health Policy and Behavioral Sciences and National Center for Sexual Violence Prevention, School of Public Health, Georgia State University, Atlanta, GA 30303, USA; agilmore12@gsu.edu

**Keywords:** adolescents, prevention, primary care, substance use, sexual assault, sexual risk, technology, mHealth

## Abstract

Primary care clinics represent a promising, yet underutilized, setting for delivering health-focused prevention programming targeting adolescent substance use, sexual assault, and sexual risk behaviors; however, little is known about adolescents’ prior exposure to such messaging. Therefore, the present study examined adolescents’ prior prevention exposure, their perceptions of prevention content, and recommendations for future programs. Semi-structured qualitative interviews were conducted with 25 adolescents (ages 14–18; 56% female, 36% male, 8% gender fluid or Two-Spirit) recruited from primary care and community settings in the southeastern United States. Thematic analysis was used to examine youth exposure to and perspectives on prevention programming. Three core themes emerged: (1) prior exposure to prevention content across topics; (2) appraisal of strengths and limitations within previous programming; and (3) recommendations for what adolescents believe their same-aged peers should know. Participants reported a preference for technology-based programs, criticized interventions exclusively promoting abstinence and negative consequences, and emphasized needing additional information on sexual consent. Adolescents in primary care settings report inconsistent exposure to prior prevention, most centering abstinence and negative consequences, rather than inclusive harm-reduction approaches. Findings highlight a structural gap in exposure to comprehensive adolescent prevention programming and position pediatric primary care as a uniquely flexible and developmentally congruent setting for delivering integrated, harm-reduction-oriented prevention interventions. These findings also further support the development of scalable, technology-driven prevention tools that can be implemented within pediatric primary care settings to improve accessibility reach, engagement, and relevance.

## 1. What Teens Hear and How They React: Adolescent Perspectives on Substance Use, Sexual Risk, and Sexual Violence Prevention in Primary Care

Substance use, sexual assault, and sexual risk behaviors among adolescents are common and interrelated (Black et al., 2011; Johnston et al., 2023; Scott-Sheldon et al., 2016). Among adolescents surveyed in the United States during 2023^1^, 42% of 12th grade students reported past-year alcohol use, 29% for cannabis, 23.2% for vaping, and 7.5% for illicit drugs other than cannabis (Johnston et al., 2023). Although these data evidence a significant decline in overall rates of substance use among adolescents—especially when compared to pre-pandemic use—the co-occurrence of substance use and sexual activity among youth is high. Globally, approximately 37% of adolescents report ever having used substances before sexual activity. Findings from national research also suggest high rates of alcohol and drug use prior to sexual activity, with 15% of adolescents reporting intoxication before their last sexual encounter (Centers for Disease Control and Prevention [CDC], 2024b). Impairments in sexual decision-making due to substance use can lead to increased likelihood of engaging in risky sexual behaviors, including earlier sexual debut, more sexual partners, inconsistent condom use, and greater rates of STIs and HIV (Cho & Yang, 2023; Noroozi et al., 2018; Patrick et al., 2020; Tapert et al., 2001). Numerous studies have also documented alarming rates of sexual violence^2^ among, suggesting between 26.6% to 53.5% of adolescents experience some form of non-consensual sexual activity (Finkelhor et al., 2014; Ngo et al., 2018), with adolescent girls (Marcantonio et al., 2022) and LGBTQ+ (Williams & Gutierrez, 2022) youth most likely to experience sexual violence. Although girls and LGBTQ+ youth experience disproportionately high rates of sexual violence (Copp et al., 2021; Pedersen et al., 2023), adolescent boys also report substantial rates of victimization (Centers for Disease Control and Prevention [CDC], 2022), underscoring the need for gender-inclusive prevention approaches. Furthermore, risk for sexual violence increases substantially within the context of alcohol and substance use (McCauley et al., 2009; Stefansen et al., 2021), highlighting the need for integrated prevention approaches that simultaneously address these intersecting risks.

Since adolescents are disproportionally affected by negative consequences resultant of substance use (Volkow & Wargo, 2022), sexual assault (Khadr et al., 2018), and sexual risk behaviors (Khreizat et al., 2025), it is critical to develop, disseminate, and implement effective prevention programs to mitigate adverse outcomes. Although substance use prevention programs are the most commonly delivered prevention interventions for adolescents (Lu et al., 2024), few adolescents receive comprehensive programming focused on the prevention of sexual assault and sexual risk behaviors (Martinez & Abma, 2010). Although School-based programming is the most common setting for adolescent prevention delivery (Das et al., 2016; Mason-Jones et al., 2016; Marseille et al., 2018), a range of systemic barriers constrain implementation within educational environments. Unlike school-based prevention programming—settings wherein strict guidelines restrain the discussion of specific sensitive content—the private, one-on-one setting of primary care clinics provide an optimal location for discussing sensitive topics such as substance use and sexual assault. Similarly, primary care clinics are also an ideal setting to screen and intervene with substance use, sexual assault, and sexual risk behavior given that most adolescents receive health prevention interventions within these settings already (National Committee for Quality Assurance, 2022); however, little is known about prevention programming adolescents within primary care settings have been exposed to. Therefore, to inform development of a primary care intervention, the present study aimed to examine previous substance use, sexual assault, and sexual risk behavior prevention programming youth in primary care settings have previously been exposed to.

### 1.1. Existing Adolescent Prevention Programming

A wide range of adolescent prevention programs targeting a diverse array of health-related behaviors have been developed and rigorously studied (Liu & Wang, 2024; Reyes et al., 2021; Saletti et al., 2021; Werner-Seidler et al., 2021). School-based prevention programs have been used widely to prevent adolescent and teen substance use (Das et al., 2016; Hennessy & Tanner-Smith, 2015), as well as prevention interventions targeting HIV, STIs, and unwanted pregnancy among adolescents (Marseille et al., 2018; Mason-Jones et al., 2016); however, existing programs targeting sexual activity often disproportionately focus on promoting abstinence (Santelli et al., 2006). While national guidelines outline standards for comprehensive sexuality education, the implementation, dissemination, and youth exposure to these interventions remains highly inconsistent across states and districts (Guttmacher Institute, 2024). Similarly, although programmatic efforts to prevent sexual violence and assault occurring among adolescents have been developed (De La Rue et al., 2014; Walsh et al., 2018), their use has been far more limited when compared to programs seeking to prevent substance use and sexual risk behaviors. While numerous programs have been developed to address substance use, sexual assault, and sexual risk behaviors, these efforts have largely occurred in isolation from one another and in school-based contexts. Similarly, even the existence of numerous studies extensively evaluating existing prevention programs for adolescents, little is known about how youth interpret and internalize prevention messages across these domains—insights essential for developing integrated prevention approaches for adolescents in primary care (Das et al., 2016; Mason-Jones et al., 2016).

### 1.2. Adolescent Exposure to Prevention Programming

In contrast to the significant attention researchers have paid to evaluating the effectiveness of existing prevention programs, comparatively little focus has been given to understanding the extent and nature of adolescents’ real-world exposure to these programs (Banyard et al., 2022). Findings from existing studies suggest that exposure to substance use prevention messages vary among adolescents, despite the widespread acceptance of teaching substance use prevention in the United States. According to a national sample of adolescents aged 12 to 17, 11.3% of adolescents reported that they were exposed to substance use prevention programs outside of school in the past year, 75% reported exposure to prevention messages in the media or at school, and 50% reported exposure to informal messaging from their parents (Lipari, 2017). Similarly, although sexually transmitted infections are on the rise and have reached an all-time high in the United States (Centers for Disease Control and Prevention [CDC], 2019), exposure to prevention efforts targeting sexual risk behaviors are variable based on state policies. In fact, while 36 states and the District of Columbia require school-based sex education, only 17 states mandate that this programming be medically accurate, 27 states emphasize abstinence-only curricula, and only 11 states require education on sexual consent (Guttmacher Institute, 2024). These findings are mirrored within national survey reports, wherein nearly all adolescent students report some exposure to STI and HIV prevention, with just over 40% of youth reporting exposure to birth control programming (Guttmacher Institute, 2022; Martinez & Abma, 2010). Students that received information about birth control methods were significantly more likely to report future and more effective contraception use, compared to those not exposed to prevention efforts (Cheedalla et al., 2020), underscoring the critical need for this content. Despite the promise of providing students with prevention programming on a range of sexual topics, most school-based sexual risk behavior prevention programs exclude information on preventing sexual assault, as well as education related to LGBTQIA+ (lesbian, gay, bisexual, trans, queer, intersex, and asexual/ally community) sexual health (Kosciw et al., 2020). In fact, these exclusions are often shaped by state-level legislative restrictions and curricular guidelines that require programs to provide education abstinence-focused content and limit information on sexual orientation, gender identity, and sexual consent, ultimately which creates further disparities in the implementation, dissemination, and exposure to prevention exposure across jurisdictions (Guttmacher Institute, 2024; National Conference of State Legislatures [NCSL], 2023).

## 2. Prevention in Primary Care Settings

Although schools are an optimal setting for prevention messages for substance use, sexual assault, and sexual risk behaviors because school attendance is mandatory for adolescents, there are significant gaps in terms of evidence-based prevention within the school system. Notably, many schools rely on programs that lack a sufficient evidence base to support their use, despite evidence documenting efficacy or effectiveness (Lynam, 2000; Lynam et al., 1999). Further, school-based prevention programming is further hindered by several systemic barriers, including legislative restrictions on discussing sexual health content (SIECUS, 2023), an overemphasis on abstinence-only approaches (Santelli et al., 2017), vague guidelines pertaining to minimum standards of existing interventions (Miedema et al., 2020), and insufficient duration to address the varied and diverse behavioral health needs of all students (Langford et al., 2014). Primary care offices are an alternate setting to screen and intervene with substance use, sexual assault, and sexual risk behaviors because it is the most likely setting for adolescents to seek preventative healthcare. The delivery of prevention interventions within primary care settings is not a novel approach. In fact, numerous prevention intervention for adolescents in primary care have been developed specifically to address psychological and behavioral health outcomes, wherein many of these programs have evidenced benefits above and beyond usual primary care outcomes (Knepper et al., 2024; Rojas et al., 2019). Researchers and adolescents alike have called for the development and implementation of adolescent-focused prevention programs addressing substance use, sexual risk, and violence in primary care settings (Tiffany-Appleton et al., 2023); yet these efforts have yet to be integrated within pediatric-focused clinical settings in a consistent way. Given that adolescents are most likely to seek preventative healthcare within primary care settings, this context represents an ideal setting to screen and intervene with these behaviors (Hofer et al., 2011).

Currently, the American Academy of Pediatrics recommends that pediatricians complete screening for substance use, brief intervention, and referral to treatment (SBIRT) with all adolescent patients using developmentally appropriate tools Levy et al. (2016). Although SBIRT is effective and easy to disseminate, the youth version is used suboptimally within adolescent primary care settings (see Mitchell et al., 2013 for a review of SBIRT’s applications for adolescents). Despite its close connection to substance use, sexual risk behaviors are assessed far less frequently in primary care settings. Similarly, while integrated brief interventions targeting concurrent substance use and sexual risk (Dermen & Thomas, 2011; Estrada et al., 2015)—and several HIV and STI prevention programs have been developed for adolescents in emergency departments and other clinical settings (Goesling et al., 2014; Miller et al., 2017)—relatively few interventions have been developed specifically for use in pediatric primary care. Existing sexual risk interventions are largely developed to provide prevention for vulnerable youth with an increased likelihood of sexual risk-related outcomes, rather than universal primary prevention (Sieving et al., 2012). Even less attention has been directed toward assessing adolescents’ histories of sexual assault victimization in primary care. For instance, in one study only 4% of adolescent primary care physicians were found to be aware of their patients’ sexual assault histories (Diaz et al., 2004), even though pediatric providers are particularly well-positioned to receive disclosures of sexual victimization and respond appropriately. Given that lack of trust is documented barrier to disclosure of sexual victimization (Münzer et al., 2016), and considering the trust often established through the continuity of care in primary care settings (Merenstein et al., 2023; Tarrant et al., 2010), delivering screenings through pediatric providers may significantly increase disclosure rates. This, in turn, may also increase the likelihood that adolescents will receive timely and appropriate post-assault interventions, potentially mitigating adverse physical and mental health outcomes associated with sexual victimization (Danielson & Holmes, 2004).

## 3. Current Study

To the best of our knowledge, no studies have assessed adolescents’ reactions to and preferences for prevention messaging and programming on substance use, sexual assault, and sexual risk behaviors within primary care settings. Therefore, more information is needed to understand what prevention-related content adolescents are exposed to in other settings (e.g., schools, media, or community programs) to inform the development of primary care-based prevention interventions. Using semi-structured qualitative interviews, the primary goal of the present study was to comprehensively understand adolescents’ experience and exposure to a variety of prevention programming across numerous settings, including their opinions on the specific programs they have been exposed to, as well to understand what they think would be important for adolescents to know about these topics. Specifically, these findings served to inform a larger study aims to develop and mHealth prevention program for adolescents in primary care settings.

## 4. Methods

### 4.1. Participants

A total of 25 adolescents aged 14 to 18 participated the present study. Of those participants, 19 adolescents completed individual interviews and were recruited from primary care clinics affiliated with an academic medical center located in the Southeastern region of the United States. Since we were unable to reach thematic saturation among sexual and gender minority adolescents while recruiting in primary care clinics, 6 additional adolescents were recruited within a community advocacy center serving LGBTQIA+ youth. Among those participants, 4 participated as part of a focus group, with the remaining two participants completing individual interviews. The average age of participants in this sample was 16.28 years old (*SD* = 1.37). A total of 56% (*n* = 14) of participants identified as female, 36% as male (*n* = 9), and 8% (*n* = 2) as gender fluid or two-spirit. A total of 44% (*n* = 11) of participants identified as Black/African American, 36% (*n* = 9) as White, 8% (*n* = 2) as Hispanic, 8% (*n* = 2) as multi-racial, 8% (*n* = 2) as other, and 4% (*n* = 1) as Native American. A total of 60% (*n* = 15) of participants identified as straight, 16% (*n* = 4) as gay or lesbian, 16% (*n* = 4) as bisexual, and 8% (*n* = 2) as pansexual. More than half of participants (56%, *n* = 14) were Medicaid recipients. To ensure that participants had relevant feedback to provide the research team on substance use, eligibility criteria for the present study required participants to: (1) be an adolescent between the ages of 14 and 18 years; and (2), endorse self- or peer-substance use in the past year. Participants were excluded if they were unable to read English proficiently or had an intellectual disability that prohibited them from understanding the content.

### 4.2. Procedure

Age-eligible participants, as indicated within a medical chart, were approached by a research assistant in the waiting room during a well visit^3^ at a pediatric office in the Southeast region of the United States. Potential participants were screened for personal or peer use of substance use, ability to read English, and intellectual disabilities that would impede the ability to complete the study procedures. If eligible, potential participants between the ages of 14 to 17, along with their parent or guardian, were invited to join the research assistant in a private room to review informed consent and assent procedures. Participants who were 18 years old were able to complete informed consent procedures without parental approval. Participants first completed a brief self-report questionnaire, followed by a semi-structured interview. Among participants completing the study within a primary care setting, the timing of study procedures was variable to accommodate clinician availability, wherein participation took place before the primary care appointment, after the primary appointment, or a combination of the two. Data collection concluded once thematic saturation was achieved. Participants were compensated $20 in gift cards for their participation. All interviews were conducted in-person, audio-recorded, and transcribed by a third-party professional transcriptionist. All study procedures were approved by the university’s Institutional Review Board.

### 4.3. Quantitative Measures

**Demographics**. Participants were asked about their age, race/ethnicity, gender, and sexuality in a self-report measure.

**Substance use.** Participants were asked to indicate alcohol use with the following questions: (1) “In the past year, on how many days have you had more than a few sips of beer, wine, or any drink containing alcohol?” (NIAAA, 2019) with an open text response and (2) “At what age did you first have more than a sip of beer, wine, wine coolers, or liquor to drink?” and response choices were “I’ve never had alcohol” or an age between 1 and 18. Participants were asked to indicate substance use with the following questions: (1) “In the past year, on how many days have you used substances or prescription drugs that were not prescribed to you?” (adapted from NIAAA, 2019 question) with an open text response and (2) “At what age did you first use a drug or prescription medication not as prescribed?” and response choices were “I’ve never used a drug or prescription medication not as prescribed” or an age between 1 and 18.

**Sexual behavior.** Participants were asked “how old were you when you first had consensual sexual intercourse (the age you chose to become sexually active)” and response options were “I’ve never had consensual sexual intercourse” or ages between 10 and 18.

**Comfort Talking to Pediatric Provider About Substance Use, Sexual Assault, and Sexual Risk Behaviors.** Participants were asked if they were comfortable having a conversation about substance use, alcohol use, smoking, sexual assault, sexual activity, contraception, pregnancy and sexually transmitted infections with a dichotomous (yes/no) response option.

### 4.4. Qualitative Interview

To inform an intervention being developed as part of a larger clinical trial examining the acceptability, usability, and feasibility of a tablet-based prevention program within primary care (Gilmore et al., 2023a, 2023b), participants were asked open-ended interview questions to gain a detailed context regarding their exposure to and experiences with previous prevention programming in any setting on the topics of substance use, sexual assault, and sexual risk, as well as their likes and dislikes of these prevention messages. Participants were also asked what other adolescents needed to know about substance use, sexual assault, and sexual risk.

### 4.5. Data Analytic Procedure

The research team included academic clinical psychologists and public health researchers with expertise in adolescent substance use, sexual violence prevention, and primary care intervention development, wherein several team members also prior experience developing and evaluating prevention programs within healthcare settings. Notably, it is possible that these backgrounds may have shaped the framing of interview questions and interpretation of findings, particularly regarding the emphasis on integration within primary care. To mitigate bias, coding was conducted independently by two trained qualitative coders, wherein regular meetings were held to challenge assumptions and further refine thematic interpretations.

Qualitative analyses were based on the approach developed by Bradley et al. (2007). The coding process was an iterative approach, allowing the coders to organize the data, resolve any emerging questions, and identify and refine thematic categories to develop key themes. A thematic analysis approach was used such that early themes shaped continued data collection until no new information was gained in subsequent interviews (i.e., thematic saturation). Thematic saturation was determined through an iterative review of emerging codes across interviews, whereas saturation was considered achieved when no new thematic categories or subcodes emerged in the analysis of consecutive interviews. Predetermined codes were developed based on questions posed to participants in the interview and refined based on results of thematic analysis. Coding discrepancies were resolved through discussion between coders, wherein disagreements were reviewed in-depth until consensus was reached. When necessary, the principal investigator served as a consensus coder, adjudicating coding decisions to refine and finalize the coding framework. Once agreement was verified, the final coding scheme was applied to the full set of study transcripts. Two coders (second and third authors) qualitatively analyzed each transcript with excellent interrater reliability (*k* = 0.97).

## 5. Results

### 5.1. Descriptive Results

Frequency of primary care visits and endorsement of past alcohol use, drug use, and sexual intercourse is provided in Table 1. Participant’s self-reported comfort in discussing numerous substance use and sexual activity-related topics with their provider(s) is presented in Table 2.

### 5.2. Qualitative Results

The finalized qualitative themes are presented in Table 3 and resulted in three thematic categories, which broadly aligned structure of the interview questions: (1) Previous Prevention Programming (substance use, sexual assault, and sexual risk behaviors), (2) Likes and Dislikes about Previous Programming, and (3) What Adolescents Need to Know about Prevention.

**Thematic Category 1: Previous Prevention Programming**. Participants were asked about exposure to previous prevention programming with regard to substance use, sexual, assault, and sexual risk behaviors. Overall, responses varied based on the programming topic they received, the method by which they received programming (i.e., location or setting), and the content presented to them.

***Substance use*.** All participants had exposure to substance use prevention programming at school, with content embedded in classes (56%, *n* = 14) or presented by school speakers (48%, *n* = 12). Participants described the content as being focused on negative consequences of substance use (72%, *n* = 18), such as effects of substances on the body, impacts on functioning, and addiction. One teen [*Male, 16, Straight, African American/Non-Hispanic*] reported the following:

*It was like we had a lesson on it, like it tells you how bad it is, about the effects of it… [Our teacher] told stories about stuff that happened [with] the kids, some of them died already but he just—we decide to do a project on it.*

Other participants (20%, *n* = 5) said their schools used more of an abstinence-based approach to substance use prevention. One teen [*Male, 16, Straight, African American/Non-Hispanic*] reported, “*They’ll teach you about [substances] and help us to tell you not to use it, all of that stuff like that.*” Another teen [*Female, 16, Straight, African American/Non-Hispanic*] also recalled being exposed to abstinence-based programming, “*[our school] actually has like a group, say like ‘Say No to Drugs’, no drinking, it’s not healthy for you and things like that*.”

***Sexual assault*.** Responses varied regarding exposure to sexual assault prevention programming, with nearly one-quarter of teens reporting no exposure (24%, *n* = 6). Of those reporting exposure, (48%, *n* = 12) identified school-based programming as the source of information about sexual assault, while the remaining adolescents (16%, *n* = 4) suggested being exposed to both informal prevention messaging and/or more formal prevention education online or on television. One participant [*Female, 14, Straight, African American/Non-Hispanic*] stated, “*Nobody really talks about that*.” Another participant [*Female, 18, Straight, African American/Non-Hispanic*] who did not receive sexual assault programming stated the following:

*None, none. I actually just started learning about sexual assault the last like two years because a group that I am in, we partner with [name of local women’s shelter] to do like, I’ve been with them that’s when I started learning more about it. But there’s really not anything in schools that will tell you about anything unless you get to college, and then they have like the whole consent thing.*

Another participant [*Female, 16, Straight, White/Non-Hispanic*] identified school as the location she received sexual assault prevention programming, though the scope of content appeared to be somewhat limited:

*I don’t think they talked about—well no, I think that there was like a one day on sort of ‘what is consent’ but like, it wasn’t really the most important thing that they cared to cover. Just kind of skipped over a bit. It was more, ‘consent is important. Get consent.’ Okay.*

When asked about the sexual assault prevention content they received, participants said they were provided with post-victimization reporting options and resources (32%, *n* = 8). One adolescent [*Female, 16, Straight, African American/Non-Hispanic*] responded as follows:

*When we talked about it, he just tells us who to contact if this happens. We should be able to talk to somebody about it. If we see or know about it going on, we should inform somebody, stuff like that.*

***Sexual risk behavior*.** More than half of participants (56%, *n* = 14) of received sexual risk prevention programming in their schools. A smaller number of participants (20%, *n* = 5) said they received this information at home, mostly through discussions with their caregivers and other family members. Teens most frequently identified that sexual risk programming and discussions were focused on topical areas such as contraception (64%, *n* = 16) and potential negative consequences of sexual activity (48%, *n* = 12), including risk for sexually transmitted infections and pregnancy. One participant [*Male, 16, Straight, African American/Non-Hispanic*] said the following:

*Oh that, all is like, it is in health class. We [were] taught about condoms or stuff like that and how to stay safe. We [were] taught about STD’s and like how to prevent it. Wear a condom every time you had sex with somebody but, that’s really all. We didn’t do any practice either. Well, our teacher, our health teacher taught us stories about it. But it wasn’t nothing like a big lesson or something.*

Another teen [*Female, 16, Straight, White/Non-Hispanic*] recalled the following:

*But the things that I know that they did cover was, you know, what is sex and, you know, they did talk briefly about some methods of contraception. There is nothing about like what do you actually do if you are pregnant or, you know, they can’t talk about abortion or things like that. And I think that they did talk about preventing STD’s and some specific STD’s and I mean things that I can remember that they talked about.*

While most teens reported some previous exposure to prevention programming and/or messaging across each of the content areas, there was significant heterogeneity in the content presented for each topic, in a range of diverse settings.

### 5.3. Thematic Category 2: Strengths & Weaknesses of Previous Programming

Adolescents were asked to reflect on the strengths and weaknesses of previous prevention programs that they had encountered, including what aspects they found most and least engaging. Additionally, participants were asked to share their opinions on gaps within the prevention programming they received.

***Strengths.*** Participants reported liking the content that they received (36%, *n* = 9), such as negative consequences associated with each risk area or information about early prevention and keeping their bodies safe. Others appreciated the style and delivery of the interventions (40%, *n* = 10), including the use of technology and the support they felt from those providing the information. One participant [*Female, 18, Straight, African American/Non-Hispanic*] liked the delivery of the content she received, noting the following:

*What I like was that they are even talking about it at our level because people seem to think that well a known stereotype is like people of a younger age are like so detached and like really don’t know anything about it or like we’re not smart enough to get it. But like the fact they even came in to talk to us was like okay, we kind of see like this is something we need to know.*

Another participant [*Female, 16, Straight, African American/Non-Hispanic*] appreciated the supportive approach her teacher provided, stating the following:

*I like that it’s not like awkward or uncomfortable. The environment is kind of friendly and it’s more so educational… Yeah. I guess it’s just the vibes like, you can tell that they actually care and not that like, oh they’re just doing their job. It’s more than that.*

Another teen [*Female, 16, Straight, White/Non-Hispanic*] mentioned the effectiveness of technology when presenting this content:

*And they used some like YouTube videos within the class when I was there which I think is helpful and more engaging than like, books and paperwork for teenagers. So that it was good that they did that.*

***Weaknesses.*** In addition to reporting strengths of previous prevention programming, participants also identified areas for improvement or that were missing. Over half (60%, *n* = 15) suggested that the content they received was not sufficient, and they would have preferred more information, activities, and resources, including more exposure to sexual assault prevention. For example, one teen [*Female, 18, Straight, African American/Non-Hispanic*] suggested that sexual assault programming focus more on creating awareness, rather than focusing on victim-focused behaviors that could increase personal risk, wherein she stated:

*I don’t like, it was more of how the information was presented. So, it was just like don’t do this, don’t see that, they are not like giving a reason why or like actual evidence of like why not to do it other than commonsense don’t do it, you know… It would just be better if people like more aware that it happens instead of then thinking oh that’s only for like the older generation because it still happened today. So, it would just be like being more aware.*

A smaller number of participants (16%, *n* = 4) of the entire sample, (30%, *n* = 3) of those identifying as sexual and/or gender minorities, expressed that relatively no emphasis is placed on the unique needs of LGBTQIA+ teens with regard to substance use, sexual assault, or sexual risk prevention programming. While only a small percentage of teens raised this issue, it is a noteworthy finding given the increased risk associated with each of these difficulties in LGBTQIA+ populations. One adolescent [*Female, 16, Straight, White/Non-Hispanic*] recalled:

*You know what, they weren’t allowed to talk about anything but heterosexual sex. And I come from—within my school there’s a pretty large LGBT community and they weren’t allowed to talk about homosexual sex or anything of this sort at all, nor abortion which they weren’t even allowed to bring it up even if you, you know, talked after class. Like it didn’t matter, they weren’t allowed to say anything about it.*

Another participant [*Male, 16, Gay, White/Non-Hispanic*] mentioned the following:

*And then I know again since moving from [name of state], I’ve had one sex-ed class in my 8th grade science and we were split, male and female. And nothing—really nothing about LGBT and such whether it’d be trans, lesbian, gay like and I even had a teacher like say like that, you know, sex is bad and like that you just shouldn’t.*

Still others raised concerns regarding the intervention delivery (36%, *n* = 9), stating that schools should provide more open, interactive discussions and this content should be better integrated into classes with all students. One participant [*Female, 17, Straight, White/Hispanic*] suggested integrating prevention programming into classes for all students:

*[Our class] only lasts a semester but it’s basically only nine weeks. Yeah and so it doesn’t last very long and I feel like they could incorporate a whole lot more if it was just a single class where you would go to every single day and, like, you could learn more about it.*

While participants highlighted a variety of content areas they liked, adolescents also suggested that programming be more inclusive of diverse identities, provide more comprehensive information as opposed to abstinence-only programming, and provide a forum to address these content areas over a longer time period, rather than simply restricted to a single class or discussion.

**Thematic Category 3: What Adolescents Need to Know about Prevention.** Adolescents were asked to share their views on what their peers should be taught about sexual assault, substance use, and sexual risk behaviors. Participant responses highlighted areas they perceived as most critical for prevention programming to address.

***Negative Consequences***. Participants overwhelmingly (100%, *n* = 25) endorsed the importance of education about negative consequences, including biological, cognitive, and behavioral impacts of substances, sexually transmitted infections resulting from risky sex, and legal consequences of initiating sexual assault. One participant [*Female, 16, Straight, White/Non-Hispanic*] addressed negative consequences of both sexual risk behaviors and substance use:

*I think that there needs to be more, I don’t know, education. I don’t even know how you’d do it, but on the dangers. Because teenagers think they’re invincible a lot of the times. And so, you know, I have friends who think it’s no big deal, it’s one time or it’s just this, it’s no big deal. And I think that a lot of teenagers especially with alcohol don’t understand like, alcohol poisoning and like people die from this. They think it’s all for fun or, you know, trying different drugs and how, oh it’s just one time. It’s no big deal but, you know, there are dangers.*

Another participant [*Female, 14, Lesbian, African-American/Non-Hispanic*] identified impacts of sexual assault on mental health:

*How to stay away from it, how to avoid it, yeah and how to see [risky situations] before it could happen. And what it does to you internal like your mental self and how you could fix it. … how it affects you.*

While most discussed negative consequences associated with sexual assault victimization, others mentioned that adolescents should be aware of boundaries and consequences associated with initiating sexual assault. One teen [*Male, 16, Straight, African American/Non-Hispanic*] said the following:

*They need to know what they can do. They need to know what it is like the definition of it, actions that considered sexual assault. The way they play like what they could do wrong, that can be sexual assault because they probably don’t know anything about it. And they’re probably doing things that they don’t really mean no harm but it can.*

***Defining Sexual Consent & Establishing Boundaries***. Teens (48%, *n* = 12) also wanted their peers to learn relevant definitions pertaining to sexual activity, consent, and establishing sexual boundaries to help facilitate education about prevention. One participant [*Female, 17, Heterosexual, White/Hispanic*] mentioned the importance of consent when asked what teens should know about sexual assault:

*I think they need to know that, like, where boundaries are. Some people think, like, oh he just touched me it wasn’t anything but that’s still part of it. I think they need to know, like, if you say no and they continue and that has already crossed the line into like sexual assault or harassment or something like that. I feel like we don’t really touch on that. And, like, especially younger students who don’t really know much about it, they need that information more than anybody else.*

Another teen [*Female, 18, Lesbian, White/Non-Hispanic*] also suggested that it is critical for prevention programming to address issues pertaining to sexual activity and consent when intoxicated:

*I think it’s important to add that substance abuse also makes it like difficult to perceive consent and to get consent or to understand that you’re not giving consent. I think that it’s important that they know that and to know that in order to have like sex safely and to understand what’s going on and to make sure that you are ready as a person, like emotionally and physically. You need to be clear headed and not be drunk or high.*

Similarly, another teen [*Two-Spirit, 18, Bisexual, Native American*] emphasized that teens should understand that consent can be revoked at any point during sexual activity:

*“What needs to really be kept clarified is consent can go away any moment. And I do think it needs to be clear also about the fact that if that consent were to go away and you not listen even if there was consent originally you can still be charged for rape.”*

***Access to Resources.*** Finally, participants also recognized the importance of supportive resources for teens (56%, *n* = 14), such as how to get help if they are struggling in any of these areas, support groups, and who to talk to following sexual assault. One participant [*Female, 16, Straight, White/Non-Hispanic*] identified a lack of resources for her peers, stating the importance of providing support for sexual assault victims:

*That’s important and there’s no—there’s no education, there’s no resources, there’s no what do you do if this happens to you, what do you do if it happens to a friend, what do you do if you think this has happened. I mean there’s nothing. There’s, tell the school guidance counselor if something’s wrong. But, you know, then people think they’re going to get in trouble for it or, you know, they don’t want to get somebody else in trouble or they don’t want to be, you know, a social leper because of it. And it’s kind of hush-hush this doesn’t really happen to—this would never happen to me, this would never happen to us. And it does, and especially going into college and leaving the safe grounds of high school which aren’t always too safe but, you know.*

Another participant [*Male, 17, Bisexual, White/Non-Hispanic*] also suggested that despite increased awareness of sensitive topics, how to access these resources is unclear:

*I see plenty of signs and posters like even driving down the highway, there is a billboard somewhere that says ‘if you’ve been assaulted you need to tell somebody.’ But it often doesn’t cover how, you know, oh go, get help or where? I mean especially, if it’s even someone close, I mean you know, the range you are looking at is 14 to 17, some of us don’t even have phones. So, how are we going to acquire the said help?*

Broadly, adolescents asserted that more comprehensive education across all topic areas, with a specific focus on negative consequences, operationalizing and defining terms pertaining to sexual activity and consent, and greater visibility of and information on accessing resources are critical knowledge areas that prevention programming address.

## 6. Discussion

The present study is among the first to qualitatively examine how adolescents perceive and react to prevention messaging about substance use, sexual assault, and sexual risk across multiple settings to inform future primary care-based interventions. Although all participants reported some exposure to substance use prevention, adolescents described this programming as being primarily abstinence-based, emphasizing the negative consequences of alcohol and drug use, rather than providing harm-reduction strategies. While abstinence-focused substance use prevention is an effective strategy for young adolescents (Smith et al., 2017), this may not be person-centered for all youth (Passetti et al., 2016)—particularly those currently using substances, who may need greater support in reducing harm related to their ongoing use (Toumbourou et al., 2007). Since adolescents who use substances are often aware of the associated risks and negative consequences of their use (Benthin et al., 1993), harm-reduction approaches may offer a viable alternative by acknowledging the perceived benefits of use, while also providing youth with behavioral strategies to reduce frequency, intensity, and adverse substance-related outcomes (Winer et al., 2022). In fact, harm-reduction approaches tailored to adolescents’ developmental level, social context, and personal preferences may empower youth to make safer choices without implicitly endorsing risky behaviors (Bonar et al., 2018; Santelli et al., 2017).

Conversely, adolescents’ exposure to programming addressing sexual assault and sexual risk behaviors was markedly less consistent, suggesting that there is a large gap in adolescent prevention programs for addressing sexual activity-related content. Several factors may contribute to variability in exposure to sex-related programmatic efforts, including significant differences in state and district-level policies regulating school-based discussions of sexual activity and gender. Participants’ observations that schools exclude discussions of LGBTQIA+ identities and certain aspects of sexual health likely reflect broader state- and district-level curricular constraints, further underscoring the potential value of primary care settings as an alternative context less constrained by legislative restrictions. National data underscore these gaps, with only 53.5% of adolescents reporting receipt of sex education aligned with national guidelines, including instruction on contraception and STI prevention (Guttmacher Institute, 2022). While nearly 90% of participants who received sexual risk content reported receiving prevention information on STIs and HIV, only 46.5% were provided information on how to access condoms, and less than 60% of adolescents received instruction on proper condom use (Guttmacher Institute, 2022). These disparities in program exposure are consistent with other national-level reports, wherein only a small minority of middle and high school students receive comprehensive programming covering all CDC-recommended sexuality topics (Centers for Disease Control and Prevention [CDC], 2024a). Findings from the present study mirror these national trends, particularly in terms of inconsistency content coverage. Based on feedback obtained within the present study, prevention messaging should also include education on the consequences of risky sexual behavior, information on available resources for sexual assault, and inclusive content addressing sexual risk and sexual assault among LGBTQIA+ youth, underscoring the importance of delivering consistent, inclusive, and evidence-based messaging on sexual health and sexual violence prevention (Black et al., 2011; Satterwhite et al., 2013).

Taken together, these findings reflect the multilevel nature of adolescent prevention processes—wherein engagement with prevention messaging is shaped not only by message content but also by the relational and contextual settings in which these messages are encountered. Participants described distinct experiences with prevention across school, peer, family, and healthcare environments, wherein they contrasted the impersonal and abstinence-focused tone of school-based messages with the greater trust and confidentiality they associated with primary care. From an ecological perspective, participant responses illuminate how multiple social contexts converge to shape receptivity to prevention messaging (Bronfenbrenner, 1979; Resnick et al., 1997). At the interpersonal level, adolescents reported learning about substance use, sexual behavior, and consent through observing those within their social environments (i.e., parents, peers), which broadly reflects the impact of observational learning and normative influences emphasized in social learning theory (Bandura, 1986). At the individual level—and consistent with developmental research on adolescent autonomy and moral reasoning (Steinberg & Cauffman, 1996)—participants also described evaluating the credibility, authenticity, and personal relevance of prevention messages, wherein the greatest engagement was noted when prevention messaging aligned with their own experiences and values.

Adolescents’ desire for additional information on defining consent, the revocability of consent within and across sexual experiences, and boundary-setting suggests that existing prevention messaging may insufficiently operationalize these concepts in developmentally accessible ways. Rather than providing abstract directives to “get consent,” adolescents appear to seek concrete behavioral guidance on how to communicate boundaries, recognize sexual coercion, and navigate sexual situations involving varying levels of intoxication. These findings highlight a critical opportunity for prevention efforts to shift from awareness-based messaging toward skills-based, scenario-driven learning that strengthens both consent communication and bystander accountability. Notably, much of the sexual assault prevention messaging described by participants centered on post-victimization resources and reporting rather than primary prevention of perpetration. This pattern reflects a broader tendency within sexual assault prevention programming to prioritize response strategies over upstream determinants of sexual violence, including normative beliefs, gender-related scripts, peer acceptability of coercion, and substance-related risk (DeGue, 2014; Flood, 2024). Primary care-based interventions may offer a unique opportunity to incorporate perpetration-focused prevention by addressing sexual boundary recognition, consent negotiation, and the effects of substance-related impairment within sexual contexts in a developmentally appropriate manner. Integrating these empirically grounded insights into primary care-based prevention design could enhance ecological fit and ensure that prevention efforts resonate across the multiple contexts in which adolescents negotiate risk and protection (Golden & Earp, 2012; McLeroy et al., 1988). Integrating these empirically grounded insights into primary care-based prevention design could improve ecological fit and ensure developmental relevance and sensitivity, ensuring that prevention efforts resonate across the multiple contexts in which adolescents negotiate risk and protection (Golden & Earp, 2012; McLeroy et al., 1988).

Findings from the present study largely support the acceptability of primary-care prevention approaches, wherein the majority reported feeling comfortable discussing topics pertaining to substance use and sexual assault with their primary care providers. Pediatric primary care settings may be a promising setting to deliver health-focused prevention programming, as adolescent well-visits are specifically designed to promote health and prevent the onset of risk behaviors, while also ensuring continuity of care between youth and their providers (Hofer et al., 2011). Unlike school-based instruction, prevention efforts in clinical settings are not constrained by state-level policies governing sexual health education, wherein providers are granted more freedom in their ability to address a broader range of sensitive health-related topics, including sexual assault and risky sexual behaviors. Regardless of adolescents’ self-reported comfort, clinicians are encouraged to provide screening and brief intervention to all youth (Levy et al., 2016), and primary care settings are well-positioned to support these efforts. Collectively, these findings point to a significant gap in adolescent prevention programming; yet findings from the present study pertaining to adolescents’ self-reported comfort discussing these topics with pediatricians, pediatric primary care may represent a promising context to deliver integrated, developmentally appropriate prevention interventions.

Adolescents also emphasized the importance of using technology to deliver prevention interventions. Within the current digital landscape—characterized by smartphones, algorithm-driven content exposure, and shortened attention cycles—mHealth approaches may offer a developmentally congruent and accessible avenue for prevention delivery that aligns with how adolescents already consume health-related information. Given the limited time available during pediatric visits, technology-based tools may be aid in implementing universal screening, brief intervention, and referral to treatment (SBIRT) protocols for adolescents presenting within primary care clinics (D’Souza-Li & Harris, 2016). In fact, technology-based interventions are particularly well-suited for adolescent populations; compared to provider-led assessments, technology-based interventions have demonstrated increased efficiency, while maintaining comparable assessment sensitivity and specificity (Harris et al., 2014, 2016). Although prior qualitative research highlights the promise of mHealth approaches for adolescent mental health (O’Brien et al., 2019), less is known about how technology can be leveraged to deliver integrated prevention messaging for substance use, sexual assault, and sexual risk behaviors. An mHealth intervention embedded within primary care could incorporate interactive, response-driven modules tailored to adolescents’ sexual orientation, gender identity, and lived experiences, while simultaneously providing confidential access to developmentally appropriate and geographically relevant resources. Technology-based platforms may also allow adolescents to explore sensitive topics—such as consent negotiation or post-assault options—anonymously, potentially reducing stigma and increasing accessibility.

The current study included a diverse group of adolescents, which also included qualitative interviews with adolescents regarding prevention messaging exposure. Despite these strengths, there are a few notable limitations of the current study. First, participants were recruited from one city in the Southeastern United States, and the sample size was small, especially within subgroups. Therefore, the findings may not reflect the experiences of adolescents across the country. Future research should include multiple clinics and a larger sample size to fully understand prevention exposure on these topics. Although the sample size was modest and included adolescents recruited from both primary care clinics and a community advocacy center, this recruitment strategy was intentional and designed to capture a broader range of prevention exposure experiences, particularly among sexual and gender minority youth. Accordingly, the findings should be interpreted as exploratory and may not generalize across adolescent age groups, clinical contexts, or community settings. Future research should also examine medical record reviews and interview pediatric primary care providers to understand if conversations about the prevention of substance use, sexual assault, and sexual risk behaviors are already occurring within primary care on a consistent basis. While a substantial minority of participants within the present study reported engaging in past-year alcohol use, with several participants acknowledging other substance use within the past year, these rates are much lower than those reported in nationally representative studies of adolescents. Therefore, research aimed at evaluating programmatic feedback exclusively among substance-using adolescents may be warranted. It is also possible that because questions delivered within the interview focused on prevention messaging exposure, the themes identified were shaped by these structured questions, wherein future research could examine adolescent perspectives using more open-ended approaches independent of specific prevention prompts. Finally, the present study focused primarily on sexual intercourse, which may not capture the broader range of non-penetrative sexual experiences that are common during adolescence (Martinez & Abma, 2010)—including experiences of sexual pressure, coercion, and unwanted touching (Finkelhor et al., 2014)—which may potentially overlook early targets for prevention and consent-focused intervention.

Although preliminary, these findings highlight a critical gap in the prevention of adolescent risk behaviors. Despite experiencing an increased risk for substance use, sexual assault, and sexual risk-taking, many adolescents lack exposure to comprehensive, evidence-based prevention programs. While currently underutilized, pediatric primary care settings offer a promising context for delivering adolescent-focused prevention interventions, with the potential to improve access among at-risk youth and reduce disparities in exposure to prevention programming. Future efforts are needed to rigorously evaluate the efficacy and long-term effectiveness of interventions implemented in primary care settings to ensure they are both relevant and responsive to the complex and evolving needs of adolescent populations.

## Figures and Tables

**Table 1:** Descriptives of Primary Care Visits, Substance Use, and Sexual Intercourse.

	*n*	*%*	*M*
**Frequency of Primary Care Visits**			
*Less than Yearly*	*2*	8	
*Once Yearly*	*15*	60	
*More than Yearly*	*7*	28	
**Alcohol Use**			
*Prior Alcohol Use*	*8*	32	
*Age of First Use (years of age)*			*14.62*
*# of Drinking Days (past-year)*			*4*
**Drug Use**			
*Prior Drug Use*	*4*	16	
*Age of First Use (years of age)*			*15.25*
*# of Substance Use Days (past-year)*			*3*
**Sexual Intercourse**			
*Prior Sexual Intercourse*	*10*	40	
*Age of First Sexual Consensual Sex*			*15.8*

**Table 2:** Comfort in Discussions with Primary Care Providers.

Topic	Total	White, Non-Latinx	Racial/Ethnic Minority	Straight, Cis-Gender	Sexual or Gender Minority	Any Substance Use
**Substance Use**						
Drug use	68%	67%	69%	62%	70%	67%
Alcohol use	68%	67%	62%	56%	80%	78%
Smoking	80%	67%	81%	75%	80%	78%
**Any Substance Use**	**84%**	**67%**	**87%**	**81%**	**80%**	**78%**
**Sexual Behaviors**						
Sexual assault	64%	55%	69%	75%	40%	67%
Sexual activity	56%	33%	62%	44%	70%	55%
Contraception	72%	67%	69%	56%	90%	78%
Pregnancy	68%	78%	62%	69%	60%	55%
STIs	80%	78%	75%	75%	80%	78%
**Any Sexual Behaviors**	**88%**	**78%**	**87%**	**100%**	**90%**	**89%**

**Table 3:** Frequency of Adolescent Interview Themes & Codes.

Theme/Code	Participants
	% (*n*)
**Exposure to Previous Prevention Programming**	
Substance Use Location or Setting	
*School Classes*	56% (*14*)
*School Speakers*	48% (*12*)
Substance Use Programming Content	
*Negative Consequences*	72% (*18*)
*Abstinence*	20% (*5*)
Sexual Assault Location or Setting	
*School*	48% (*12*)
*No Programming*	24% (*6*)
*Online/Television*	16% (*4*)
Sexual Assault Programming Content	
*How to Get Help*	32% (*8*)
Sexual Risk Behaviors Location or Setting	
*School*	56% (*14*)
*Home*	20% (*5*)
Sexual Risk Behaviors Programming Content	
*Contraception*	64% (*16*)
*Negative Consequences*	48% (*12*)
**Feedback on Previous Programming**	
Strengths	
*Content*	40% (*10*)
*Style and Delivery*	36% (*9*)
Weaknesses	
*Insufficient Information*	60% (*15*)
*Delivery*	36% (*9*)
*Little Emphasis on LGBTQIA+ Needs*	16% (*4*)
**Recommendations for What Adolescents Need to Know** **About Substance Use, Sexual Assault, and Sexual Risk**	
*Negative Consequences*	100% (*25*)
*Defining Consent & Establishing Boundaries*	48% (*12*)
*Supportive Resources*	56% (*14*)

## Data Availability

The data presented in this study are not publicly available due to privacy and ethical restrictions related to the sensitive nature of the qualitative data and the protection of participant confidentiality.

## Notes

1 These data were obtained from **Monitoring the Future** (Miech et al., 2024 ), which is a nationally representative survey of nearly 25,000 students in 8th, 10th, and 12th grades across approximately 400 public and private secondary schools. The full 2023 report can be obtained using the following: https://monitoringthefuture.org/wp-content/uploads/2023/12/mtf2023.pdf (accessed on 15 March 2026).

2 ***Sexual violence*** is an umbrella term encompassing a range of non-consensual sexual behaviors, including sexual harassment, coercion, and assault (Centers for Disease Control and Prevention [CDC], 2022), whereas ***sexual assault*** typically refers to unwanted sexual contact or penetration obtained through force, coercion, or incapacitation (U.S. Department of Justice [DOJ], n.d.). Given that the present study primarily examined adolescents’ exposure to messaging related to unwanted sexual contact, we use the term sexual assault when referring to these specific behaviors.

3 A “***well visit***” refers to a routine preventive healthcare appointment conducted in paediatric primary care settings, which is typically focused on conducting age-appropriate developmental screenings and surveillance, health promotion and education, risk-based guidance, and behavioral assessments (American Academy of Pediatrics [AAP], 2019).
